# Changes in Physiological Tendon Substrate Stiffness Have Moderate Effects on Tendon-Derived Cell Growth and Immune Cell Activation

**DOI:** 10.3389/fbioe.2022.800748

**Published:** 2022-02-28

**Authors:** Subhajit Konar, Scott M. Bolam, Brendan Coleman, Nicola Dalbeth, Sue R. McGlashan, Sophia Leung, Jillian Cornish, Dorit Naot, David S. Musson

**Affiliations:** ^1^ Department of Nutrition and Dietetics, University of Auckland, Auckland, New Zealand; ^2^ Department of Surgery, University of Auckland, Auckland, New Zealand; ^3^ Department of Orthopaedics, Middlemore Hospital, Auckland, New Zealand; ^4^ Department of Medicine, University of Auckland, Auckland, New Zealand; ^5^ Department of Anatomy and Medical Imaging, University of Auckland, Auckland, New Zealand

**Keywords:** tendon, ECM–extracellular matrix, inflammation, stiffness, tendinopathy

## Abstract

Tendinopathy is characterised by pathological changes in tendon matrix composition, architecture, and stiffness, alterations in tendon resident cell characteristics, and fibrosis, with inflammation also emerging as an important factor in tendinopathy progression. The sequence of pathological changes in tendinopathy and the cellular effects of the deteriorating matrix are largely unknown. This study investigated the effects of substrate stiffness on tendon-derived cells (TDCs) and THP-1 macrophages using PDMS substrates representing physiological tendon stiffness (1.88 MPa), a stiff gel (3.17 MPa) and a soft gel (0.61 MPa). Human TDCs were cultured on the different gel substrates and on tissue culture plastic. Cell growth was determined by alamarBlue™ assay, cell morphology was analysed in f-actin labelled cells, and phenotypic markers were analysed by real-time PCR. We found that in comparison to TDCs growing on gels with physiological stiffness, cell growth increased on soft gels at 48 h (23%, *p* = 0.003). Cell morphology was similar on all three gels. SCX expression was slightly reduced on the soft gels (1.4-fold lower, *p* = 0.026) and COL1A1 expression increased on the stiff gels (2.2-fold, *p* = 0.041). Culturing THP-1 macrophages on soft gels induced increased expression of IL1B (2-fold, *p* = 0.018), and IL8 expression was inhibited on the stiffer gels (1.9-fold, *p* = 0.012). We also found that culturing TDCs on plastic increased cell growth, altered cell morphology, and inhibited the expression of SCX, SOX9, MMP3, and COL3. We conclude that TDCs and macrophages respond to changes in matrix stiffness. The magnitude of responses measured in TDCs were minor on the range of substrate stiffness tested by the gels. Changes in THP-1 macrophages suggested a more inflammatory phenotype on substrates with non-physiological stiffness. Although cell response to subtle variations in matrix stiffness was moderate, it is possible that these alterations may contribute to the onset and progression of tendinopathy.

## Introduction

Tendons have a highly aligned and hierarchically organised extracellular matrix (ECM) (Andarawis-Puri et al., 2015), primarily composed of type-I collagen fibres interspersed with proteoglycans and minimal amounts of other matrix proteins and glycoproteins (Riley et al., 1994; James H; Wang, 2006). The tissue is predominantly avascular (Petersen et al., 2000) and sparsely populated with fibroblast-like cells residing between the aligned collagen fibres (Whittaker and Canham, 1991). Tendon disease or injury leads to pain and reduced function, commonly referred to as ‘tendinopathy’, and causes characteristic changes in tendon tissue such as hypercellularity, increased vascularity and innervation, and altered matrix architecture. Although tendinopathy is common and increasing in prevalence (James et al., 2008; Clark et al., 2020), treatment options are limited and often fail to reverse the tendinopathic changes and fully restore tendon functionality. The sequence of pathological changes that occur in tendinopathy, and the interplay between the deterioration of the ECM and the cells, are still not well understood.

The altered ECM composition and structure in tendinopathy results in tendons with inferior mechanical properties, which are prone to failure (Kannus and Józsa, 1991; Millar et al., 2015; Zhu et al., 2020). Pathological changes in tendon stiffness can take one of two forms: reduced stiffness, caused by the disruption of the organised ECM structure, or greater stiffness, seen in fibrotic or calcified tendon (Evans and Barbenel, 1975; Kannus and Józsa, 1991). Tendinopathy also affects the cellular component of the tendon. Resident fibroblast-like cells increase in number and assume a more rounded shape, and inflammatory cells infiltrate the tissue (Dakin et al., 2015; Stolk et al., 2017). In the early stages of tendinopathy there is evidence of macrophage infiltration (Millar et al., 2010; Dakin et al., 2015) and increased expression of inflammatory markers, including interlukin-1β (IL-1β) (Stolk et al., 2017) and prostaglandin-E2 (PGE-2) (Fu et al., 2002; Dakin et al., 2012). Macrophages appear to contribute to the healing process, as higher levels of anti-inflammatory macrophage markers post-surgery is associated with better healing outcome (Dakin et al., 2015).

Studies in matrix biology have demonstrated that the ECM microenvironment plays a key role in regulating the activity of cells. Substrate stiffness plays a crucial role in regulating cell growth, morphology (Mih et al., 2012; Skardal et al., 2013), migration (Sridharan et al., 2019), stem cell differentiation (Engler et al., 2006), and maintaining cell phenotype (Chaudhuri et al., 2014). In tendon biology, the effect of substrate stiffness on tendon progenitor cells has been extensively studied, demonstrating that stiffness can modulate tendon progenitor cell proliferation and tenogenic potential (Sharma and Snedeker, 2010; Islam et al., 2017; Liu et al., 2018; SJ et al., 2018) Substrate stiffness has also been demonstrated to affect macrophage morphology, proliferation, and activation, however, these have typically been carried out in the low kPa range, below that of physiological tendon (Adlerz et al., 2016; Sridharan et al., 2019; Xing et al., 2021). The effect of substrate stiffness on resident tendon cells has not been well characterized. A recent study exposed human tendon cells derived from a single donor to different substrate stiffness conditions (Ryan et al., 2021). However, the effect of substrate stiffness was not the primary focus of this study as it was exploring multiple combined physicochemical cues for maintaining tendon cell phenotype.

Most studies of TDCs *in vitro* have used very stiff tissue culture plastic (TCP)to better understand tendon cell biology, which is well known to induce TDC dedifferentiation (Taylor et al., 2009). Also, the majority of current tissue engineering approaches aim to apply material scaffolds that do not match the native tendon biomechanical properties, thus creating a mismatch in properties between the intervention and tendon resident cells (Vasiliadis and Katakalos, 2020; Mao et al., 2021). Therefore, understanding how ECM microenvironemntal cues affect cell behaviour in tendon has importance for creating more physiologically-relevant *in vitro* platforms to study tendon cell biology, and for infomring future therapeutics which can better direct and maintain resident tendon cell characteristics.

Thus, the aim of the current study was to investigate the effects of substrate stiffness on resident tendon cells and on the phenotype of the infiltrating macrophages. We developed an *in vitro* gel-based platform with tuneable substrate stiffness and studied the effect of physiological and non-physiological substrate stiffness on human TDCs and THP-1 macrophages.

## Materials and Methods

### Isolation of Human Tendon Derived Cells (TDC)

Healthy hamstring and biceps tendons were isolated from patients undergoing orthopaedic surgery (Supplementary Table S1). Ethical approval for the study was obtained from the New Zealand Northern Health and Disability Ethics Committee (approval number NTX/05/06/058/AM15), and all participants provided written informed consent prior to surgery. TDCs were isolated as previously described (Chhana et al., 2014; Musson et al., 2015). Briefly, tendon tissue harvested during surgery was collected, cut into small pieces (∼1 mm^3^ size), and incubated in digestion medium (DMEM/F12, 10% fetal bovine serum (FBS), dispase (0.5 mg/ml) (all from Gibco™, ThermoFisher Scientific Inc.), and collagenase II from *Clostridium histolyticum* (400 units/ml; Sigma Aldrich) in a shaker incubator at 37°C overnight. Following digestion, cells were strained using a 70 μm cell strainer, resuspended in fresh DMEM/F12 with 10%FBS, seeded in T-75 tissue culture flasks, and incubated at 37°C/5%CO_2_. Cells were harvested at confluence and transferred to liquid nitrogen storage. Prior to use, passage 1 TDCs were revived and cultured to confluence in T-75 flasks, and then seeded on substrates of various stiffness.

### Preparing Poly-Dimethyl Siloxane (PDMS) Substrates

Poly-dimethyl siloxane (PDMS) resin and the crosslinking agent (Sylgard 184, Dow Corning) were mixed in various ratios to prepare mixtures with different crosslinking concentration. It has been previously established that PDMS stiffness can be controlled by the concentration of the crosslinking agent (Wang et al., 2014), with increased concentrations producing stiffer gels. For the present study we prepared gels of three different weight ratios (PDMS: crosslinker): 5:1 for a stiff substrate, 15:1 for a physiological-stiffness substrate, and 80:1 for a soft substrate. The reagents were mixed thoroughly, degassed under vacuum for 30 min and poured into wells of 24-well plates. The plates were incubated at 80°C for 2 h and cooled to room temperature overnight. Prior to use, the gels and the TCP substrate were functionalized with plasma treatment at 800mTorr for 2 min in 45 W Air-plasma (Harrick Plasma), coated with 0.15 mg/ml collagen-I solution overnight at 4°C, and sterilized by UV light for 30 min (Hisey et al., 2021).

### Substrate Elastic Modulus Characterization

Bovine superficial digital flexor tendons, sourced from Wilson Hellaby-Auckland Meat Processor Ltd., were cut into 1 cm^2^ pieces of sectioned longitudinally along the direction of the fiber alignment to 300 µm thickness in a cryo-microtome. The elastic modulus of PDMS gels and the bovine tendon were measured using Asylum Research Atomic Force microscope (AFM) with SiNi tips (BudgetSensor, sini-10, Silicon Nitride tips, stiffness 0.27 N/m and freq 30 kHz).

The elastic modulus measurement for all the slices and PDMS gels were conducted in an aqueous environment while hydrated in phosphate buffered saline. Prior to each experiment the tip was calibrated for stiffness and Inverse Optical Lever sensitivity in air and liquid environment. At least 30 indentations were performed at randomly chosen locations on the healthy, control bovine tendon slices (*n* = 3) and each PDMS stiffness gel group (*n* = 3–5) and were analyzed using the Asylum Research software (version 16.26). The force measurement in AFM is calculated by multiplying tip deflection with spring constant of the tip. The distance travelled by the tip was measured from the movement of the Piezo actuator height (Z). The indent portion of the curve was fitted into the hertz model (the tip assumed to have a pyramid shaped and the Poisson’s ratio (υ) of the material assumed to be 0.5) to calculate the elastic modulus of the substrates. Hertz’s contact stiffness measurement equation for micro-scale elastic modulus measurement in AFM using a pyramidal tip takes the following form

$$F=\delta^{2}\times \left(E\times \frac{\tan\left(\alpha \right)}{\sqrt{2}\times \left(1-\upsilon^{2}\right)}\right)$$

In the equation, E denotes the Young’s Modulus of the substrate, F denotes the indention force on the tip, α denotes the half angle of the pyramidal tip (half cone angle specified by the manufacturer as 35°), δ denotes the deformation of the tip, υ is Poisson’s ratio.

### Cell Growth Assay

Cell growth assays were carried out as previously described (Musson et al., 2015), using alamarBlue™ (Invitrogen™, ThermoFisher Scientific Inc.) as a measure of cell number (De Fries and Mitsuhashi, 1995; Al-Nasiry et al., 2007). Human TDCs were seeded in 24-well plates (Greiner BioOne, Sigma Aldrich) in DMEM/F12 with 5%FBS at a density of 25,000 cells/well and incubated at 37°C/5%CO_2_. The effect of substrate stiffness on TDC growth was quantified at 24 and 48 h. To quantify the cell growth, 5% alamarBlue™ (v/v) was added to the wells and incubated for 4 h at 37°C/5% CO_2_. At the end of the incubation period, 200 µl of alamarBlue™-containing conditioned medium from each well was transferred into a 96-well plate (Greiner Bio-One, Sigma Aldrich), and fluorescence determined in Synergy 2 multi-detection microplate reader (BioTek Instruments, Inc., Winooski, VT). Following the first time point, medium in each well was refreshed and the assay was repeated at 48 h. The fluorescence background reading was subtracted, and the results were normalized to the that of cells on physiological stiffness after 24 h.

### Morphological Characterization of TDC

TDCs were seeded at a density of 10^4^ cell/well in 24-well plates with the PDMS gels representing physiological, stiff and soft stiffness and incubated in DMEM/F12 with 5% FBS for 24 h. Cells were fixed in 4% paraformaldehyde for 20 min, and permeabilised with 0.5% triton-X overnight. For actin cytoskeleton staining, cells were incubated overnight with Alexa-Fluor™ 594 Phalloidin (Invitrogen™, Thermo Fisher Scientific), following the manufacturer protocol. Cells were then incubated for 3 h with DAPI (4′,6-Diamidine-2′-phenylindole dihydrochloride, Sigma Aldrich) for nuclear staining. Images of the fixed and stained cells were captured using Olympus CKX53 inverted fluorescence microscope using Olympus DP-72 camera. Cell periphery was manually traced from the images to calculate cell area, aspect ratio (the ratio between the major axis and the minor axis of the cells, AR) and circularity (defined as $\text{Circularity}=4\times \pi \times \left(\frac{\text{Area}}{{\text{Perimeter}}^{2}}\right)$ . More than 100 cells were captured per experimental group, taken from 10 images across 5 replicate wells in 3 independent biological experiments. Quantitative analysis of cell morphology was carried out using the NIH-ImageJ software (http://rsb.info.nih.gov/ij/).

### Macrophage Response to Stiffness

Cells of the human monocytic cell line, THP-1, were used as a model for macrophages. THP-1 cells, cultured in RPMI-1640/10%FBS (Gibco™, ThermoFisher Scientific Inc.), were seeded at a density of 1.5×10^6^ cells/well in 24-well plates containing the PDMS gels representing physiological, stiff and soft stiffness. Phorbol 12-myristate 13-acetate (PMA) (200 ng/ml) was added to the medium for 24 h to induce the differentiation of THP-1 cells to macrophages (Starr et al., 2018), confirmed by the cells becoming adherent and CD68^+^. The PMA supplemented media was removed after 24 h and replenished with RPMI-1640/5%FBS media. Conditioned media was collected after 48 h, and cells were trypsinised and cell pellet were collected for gene expression analysis.

### Analysis of Gene Expression by Real-Time PCR

RNA was extracted from TDCs and THP-1 cells after 48 h of culture on PDMS gels representing physiological, stiff and soft stiffness using the RNeasy^®^ Mini kit (QIAGEN). RNA was extracted following the manufacturer’s protocol, and on-column DNase digestion with the RNAse-Free DNase Set (QIAGEN) was used to eliminate DNA contamination. The RNA concentration and purity were measured using Nano-Drop Lite spectrophotometer (Thermo Fisher Scientific) with 260/280 absorbance value > 1.8 considered as acceptable. cDNA was synthesised with Superscript-III (Thermo Fisher Scientific). Gene expression was analysed in QuantStudio™ 5 Real Time PCR System, using multiplex PCR with FAM-labelled TaqMan™ assays for the target genes and VIC-labelled TaqMan™ assay for 18S rRNA, used as the endogenous control (Thermo Fisher Scientific). The relative expression level of the genes compared to cells cultured on the physiological stiffness PDMS gels was calculated by the 2^-∆∆Ct^ method. The genes studied are listed in Supplementary Table S2.

### Analysis of Macrophage-Secreted IL-1β by ELISA

Secreted IL-1β was quantified in conditioned media samples collected from THP-1 cells using DuoSet ELISA (R&D Systems) and following the manufacturer’s protocol. Absorbance of each well at 450 nm was read using Synergy 2 multi-detection microplate reader (BioTek Instruments, Inc., Winooski, VT).

### Statistical Analysis

GraphPad Prism 8.2.1 (GraphPad Software) was used for all statistical analysis. Data was assessed for normality using the Shapiro-Wilk test. Normally distributed data were analysed using either one-way analysis of variance (ANOVA) or two-way ANOVA, with post-hoc Dunnett’s or Tukey’s test. Tests were 2-tailed, and a 5% significance level was maintained throughout the study. For non-normal data Kruskal–Wallis test was performed.

## Results

### Substrate Elastic Modulus Characterization of PDMS Gels

**FIGURE 1:**
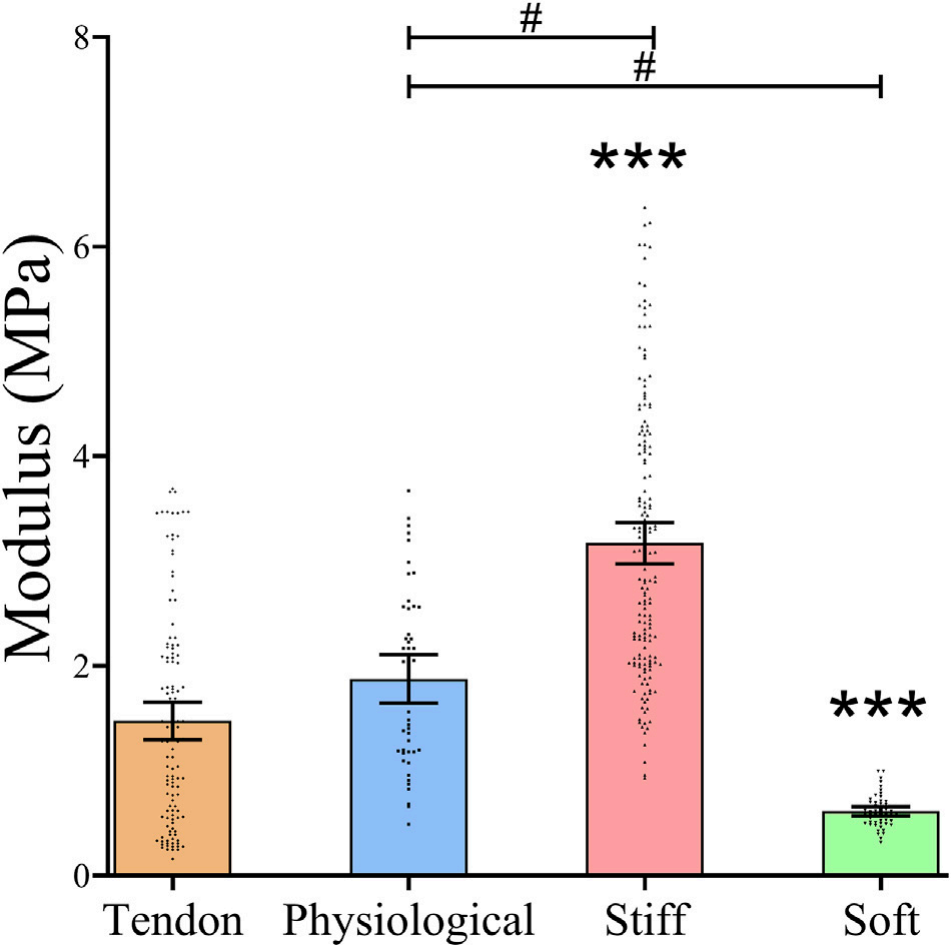
Stiffness of Tendon slices and three formulations of PDMS gels The stiffness of the matrices was measured using AFM. Dots represent individual measurements, means and 95%CI are indicated. Stiffness of the different substrates was compared by Kruskal Wallis test. * = statistically significant compared to bovine tendon slices, # = statistically significant between gel systems, both *p* < 0.05.

The mean elastic modulus of the tendon samples was 1.48 MPa (95% CI 1.30, 1.66). The elastic modulus of the physiological gel was similar to that of tendon, with a mean of 1.88 MPa (95% CI 1.65, 2.11) (*p* = 0.11). The stiff gel had a 2.1-fold higher elastic modulus (3.17 MPa; 95%CI 2.98,3.37), and the soft gel had a 2.5-fold lower elastic modulus (0.614MPa; 95%CI 0.57, 0.66) than that of the tendon (*p* < 0.0001) (Figure 1).

### TDCs Growth Is Induced on Substrates With Non-physiological Stiffness

**FIGURE 2:**
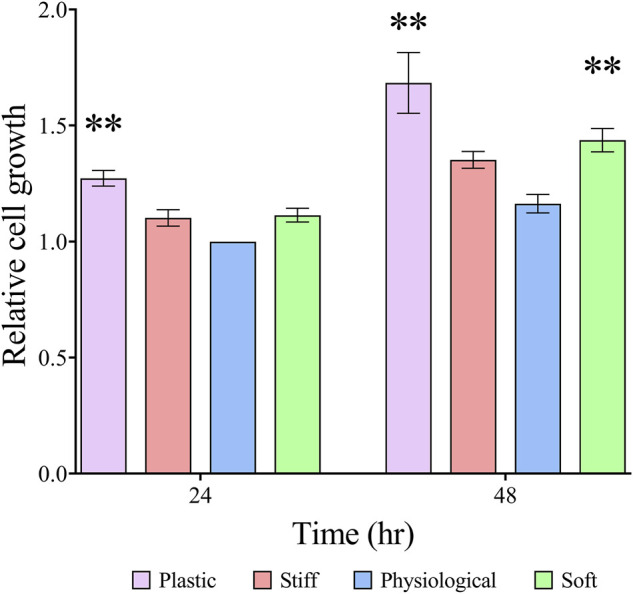
TDC growth on substrates with different stiffness Cell growth was determined by alamarBlue™ assay. Results are presented as means ± SEM (*n* ≥ 4) normalized to 24 h physiological stiffness group. Groups were compared by two-way ANOVA with post-hoc Dunnett’s test. **p < 0.01, in comparison to cell growth on substrate with physiological stiffness of the respective day.

At 24 h, TDC growth was similar on all three gel substrates and 26% greater (*p* = 0.0005) on TCP than on the physiological-stiffness gel (Figure 2). At 48 h, cell growth was approximately 23% greater (*p* = 0.003) on soft substrate, and 45% greater (*p* < 0.0001) on TCP than on the physiological-stiffness gel. To examine whether the response to substrate stiffness in our experimental system is a characteristic of the TDCs or a more general cellular response, we repeated the experiment with mouse osteoblast-like MC3T3-E1 cells. The growth of MC3T3-E1 cells was similar on TCP, physiological, and soft gels, but significantly higher on the stiff gel (Supplementary Figure S1).

### TDC Morphology is Similar on the Three gel Substrates but Different on TCP.

**FIGURE 3:**
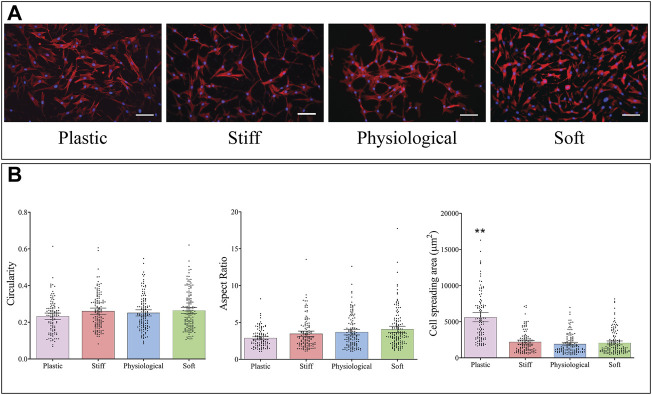
Morphology of TDCs cultured on substrates of different stiffness. **(A)** Representative images of cells stained with DAPI (blue, nucleus) and Alexa-Fluor 594 Phalloidin (red, actin filaments) (Scale bar = 100 µm). **(B)** Morphological parameters of TDCs–circularity, aspect ratio, and area were measured in cells on the different substrates. Aspect ratio; higher values indicate a more elongated cell shape. Dots represent individual measurements, means and 95% CI are indicated. Groups were compared by Kruskal Wallis test. **p < 0.01in comparison to substrate with physiological stiffness.

Cells were stained with DAPI and phalloidin (Figure 3A), and cell morphology was analysed from microscopic images. The mean area of TDCs cultured on TCP was almost three times higher than that of cells cultured on physiological-stiffness gel (*p* < 0.0001) (Figure 3B). There were no significant differences in cell morphology between cells cultured on the stiff, physiological, and soft gels.

### Gene Expression in TDC Cultured on the Different Substrates

**FIGURE 4:**
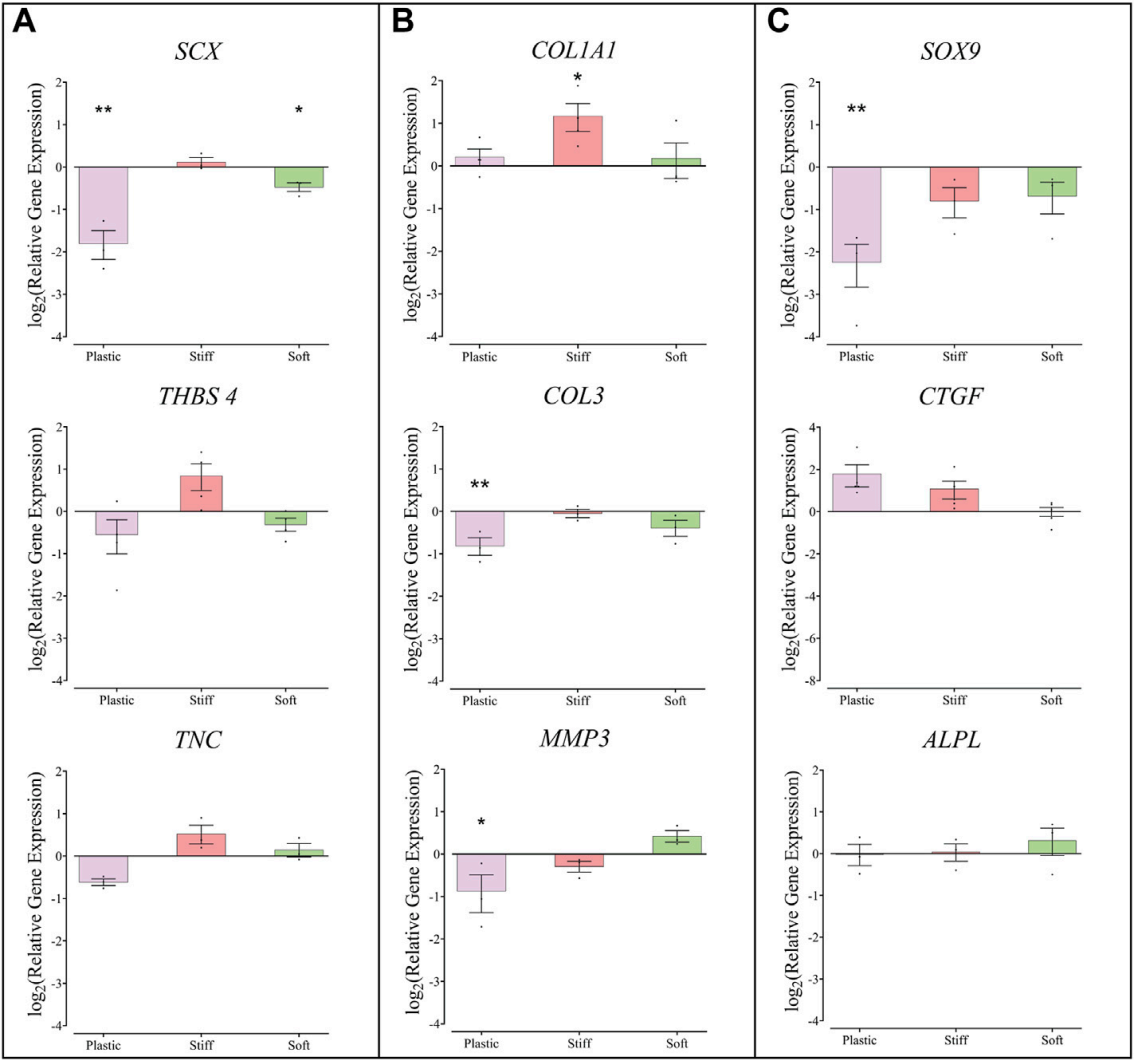
Gene expression in TDCs cultured on substrates of different stiffness. **(A)** Genes associated with tendon phenotype, Scleraxis, Thrombospondin-4, Tenascin-C. **(B)** Genes associated with matrix remodeling: Collagen-1a1, Collagen-III, Matrix metalloproteinase-3. **(C)** Genes associated with transdifferentiation and fibrosis; SOX-9, Connective tissue growth factor, Alkaline phosphatase. Expression levels are presented relative to the expression in cells cultured on the substrate with physiological stiffness (represented here by a line at *y* = o) Each dot represents one biological repeat, means ± SEM are indicated. Groups were compared by one-way ANOVA with post-hoc Dunnett’s test. *p < 0.05, **p < 0.01 in comparison to substrate with physiological stiffness (*n* ≥ 3).

Compared to TDCs on physiological stiffness substrate, cells cultured on the soft gel had lower expression of *SCX* (mean ± SEM, 0.72 ± 0.05, *p* = 0.016), and TDCs cultured on the stiff substrate had higher expression of *COL1A1* (mean ± SEM, 2.25 ± 0.50, *p* = 0.0410) (Figure 4). TDCs cultured on TCP had lower expression of *SCX*, *COL3*, *MMP3*, and *SOX9* compared to TDCs on physiological stiffness substrate. The expression of *THBS4*, *ALPL,* and *CTGF* in TDCs was similar on all substrates.

### The Effect of Substrate Stiffness on Macrophages

**FIGURE 5:**
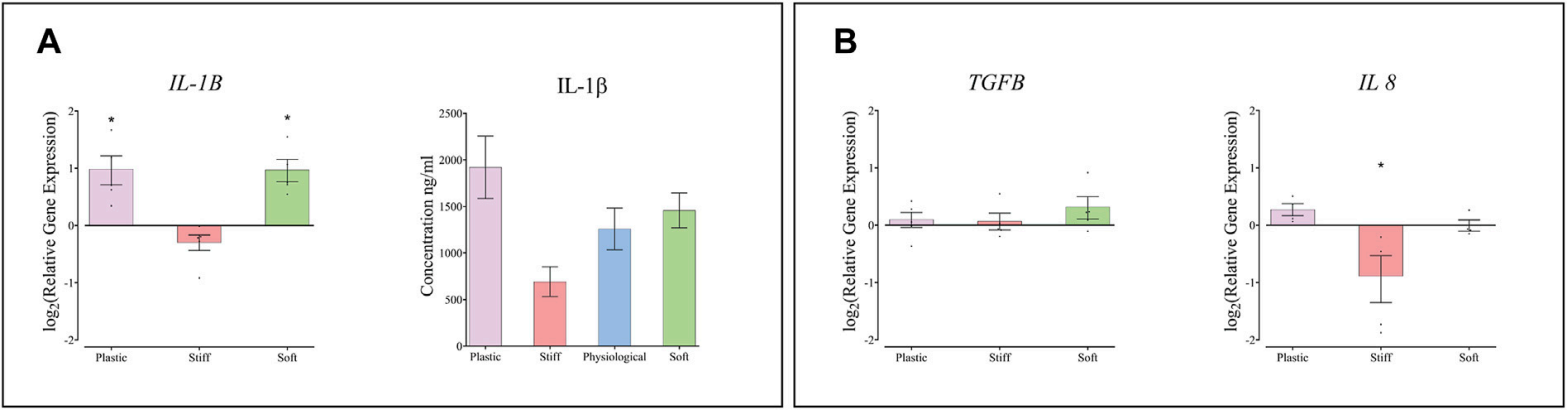
The effect of substrate stiffness on THP-1 macrophages. **(A)** Relative expression of IL1B mRNA and the concentration of IL1β protein secreted from THP-1 macrophages into the condition medium. **(B)** Relative expression of TGF and IL8 in THP-1 macrophages cultured on substrates of different stiffness. Gene expression levels are presented relative to cells cultured on substrate with physiological stiffness. Each dot represents one biological repeat, means ± SEM are indicated. Groups were compared by one-way ANOVA with post-hoc Dunnett’s test. *p < 0.05 in comparison to substrate with physiological stiffness (*n* ≥ 4).

The expression of IL1B, encoding the inflammatory cytokine IL-1β, was approximately 2-fold higher in THP-1 macrophages cultured on the soft substrate and on TCP than on the physiological-stiffness gel (Figure 5A). However, the concentration of secreted IL-1β protein in the conditioned media did not change significantly between cells cultured on the different substrates. The expression levels of IL8 and TGFB1, were also determined in the THP-1 cells. IL8 showed approximately 2-fold decrease in THP-1 cells cultured on the stiff substrate compared to the physiological substrate (Figure 5.B), whereas the expression of TGFB1 was similar on all substrates.

## Discussion

Our study found that TDCs and THP-1 macrophages respond to changes in substrate stiffness. Using a PDMS gel based *in vitro* system, we prepared substrates with physiological tendon stiffness, and two formulations that represented matrix stiffness in non-physiological condition: soft gels with 2.1-fold lower stiffness, and stiff gels with 2.5-fold higher stiffness. In comparison to TDCs culturing on physiological substrate stiffness, we found increased cell growth and reduced expression of *SCX* in TDCs cultured on the soft gels, and increased expression of *COL1A1* in TDCs cultured on the stiff gels. Culturing THP-1 macrophages on soft gels induced the expression of *IL1B*, whereas *IL8* expression was inhibited on the stiffer gels. We also found that culturing TDCs on tissue culture dishes induced increased cell growth and altered cell morphology and the signature of gene expression in comparison to the substrate with physiological stiffness, confirming that TCP is not suitable for maintaining tendon cell characteristics. Our findings suggest that changes in tendon matrix stiffness during tendinopathy play a role in altering resident cell behaviour. These alterations in cell behaviour are minor, but may contribute to the onset and progression of tendinopathy.

Although it is well established that tendinopathy affects tendon matrix stiffness, the exact magnitude of these changes at the substrate stiffness level, and their contribution to the progression of tendon disease, are still largely unknown. The tensile modulus of tendon tissue, however, has been well characterised by elastography or *ex-vivo* tensile testing (Coombes et al., 2018; Finnamore et al., 2019). In tendinopathy the tensile modulus of tendon is reduced, resulting in a less stiff tendon (Wiesinger et al., 2020). Higher stiffness characteristic of scar tissue is also relevant to tendinopathy, but our current knowledge is mostly based on measurements of scar formation in other tissues (Liu et al., 2010). Here, using AFM we determined tendon substrate stiffness at ∼1.5 MPa, which is similar to a previous study that reported the substrate stiffness of hydrated collagen fibres in tendon at 1.2 MPa (Grant et al., 2008). In the current study, we used PDMS gels as a biomimetic platform for substrate stiffness, with three different proportions of PDMS: crosslinker used to prepare stiff, physiological, and soft substrates for cell cultures. Because the magnitude of change in diseased tendon stiffness is not clearly established, we chose to model changes of approximately 2-fold change in matrix stiffness.

The main substrate stiffness-dependent effect in TDCs was a small increase in cell growth on a softer matrix, with no effect seen in TDCs cultured on the stiffer matrix. Hypercellularity is one of the main cellular hallmarks of tendinopathy (Kannus and Józsa, 1991). Our results suggest that the softer tendon matrix in tendinopathy may contribute to the increase in cell number. Interestingly, most previous studies found that stiffer substrates promote greater increases in cell growth compared to softer substrates (Evans et al., 2009; Hopp et al., 2013), similar to what we observed in osteoblast-like MC3T3-E1 cells. This suggests that cells vary in their responses to substrate stiffness, and this response is likely dictated by the physiological stiffness of their natural tissue environment. Similar to previous research, we found that TCP, a substrate with a very high stiffness in comparison to physiological tendon matrix, increased cell growth (Mih et al., 2012; Skardal et al., 2013). While the mechanisms driving the observed changes in TDC growth were not explored as part of this study, there is evidence that cell response to stiffer substrates tend to be driven by Rho-ROCK pathways (Paszek et al., 2005; Mih et al., 2012; Sridharan et al., 2019; Doss et al., 2020). Neural cells, meanwhile, have been shown to respond to softer substrates through the EGFR/PI3K/AKT pathway (Zhang et al., 2020). Understanding the mechanisms regulating TDC response to substrate stiffness could play an important role in directing future therapeutics for tendinopathy. Although there was stiffness-dependent effect with TDC cell growth, TDC morphology was comparable on the three different stiffness gels. Previous studies in this stiffness range have mixed results, with culture on stiffer substrates resulting in increased cell area and AR in human dermal fibroblasts, but no stiffness-dependent effect observed in embryonic stem cells (Evans et al., 2009; Hopp et al., 2013). *In vitro*, TDC morphology is known to be regulated by substrate architecture (English et al., 2015; Fotticchia et al., 2018; Mohammad et al., 2020). Whereas, *in vivo*, native cells elongate and align along the collagen fibres (James et al., 2008), thus changes in cell morphology that occur during tendinopathy are more likely a result of the disorganised tendon ECM, rather than changes in matrix stiffness. TDCs cultured on TCP tissue culture plastic had increased area compared to the cells on physiological stiffness gels, similar to previous studies (Mih et al., 2012; Skardal et al., 2013).

Minor effects on TDC gene expression profiles were also observed on different stiffness gels. In comparison to TDCs on physiological stiffness gels, TDCs on soft gels had lower expression of *SCX*, which encodes for a transcriptional factor essential for determining tendon cell fate. Previous studies have shown decreased *SCX* expression in tendinopathy (Taylor et al., 2009), and it is possible that lower substrate stiffness may contribute to this change. In line with previous studies, we observed higher *COL1A1* expression in TDCs cultured on the stiff substrate (Islam et al., 2017). *COL1A1* encodes for the major component of type I collagen, which is the main structural protein of tendon (Riley et al., 1994), suggesting stiffer, fibrotic regions in tendon matrix may promote further matrix production. Interestingly, we did not observe any changes in COL3A1 or CTGF expression on the stiffer substrates, which would be expected as both are associated with fibrotic healing in tendon (Riley et al., 1994; Morita et al., 2016; Nichols et al., 2019). TDCs cultured on TCP substrate had lower expression levels of a number of genes investigated, similar to previous studies that demonstrated a drift in tendon cell phenotype, and loss of tendon-selective gene expression in cells cultured on plastic (Yao et al., 2006; Jelinsky et al., 2010).

Understanding the effect of matrix stiffness in immune cell response is important in the context of tendinopathy, with histological studies observing immune cell invasion into the diseased tendon (Kannus and Józsa, 1991; Cetti et al., 2003; Abate et al., 2009). Our data indicate that exposure to softer substrate gels results in higher IL1β expression in macrophages, while exposure to stiff substrate gels results in lower IL8 expression, indicating a pro-inflammatory shift on response to non-physiological stiffness. Other studies have reported similar pro-inflammatory activation of macrophages on soft substrates, although this effect appears to be reversed in the presence the biochemical mediator LPS (Previtera and Sengupta, 2015; Chen et al., 2020; Chuang et al., 2020). There is emerging evidence that inflammation plays a role in the progression of tendinopathy (Abate et al., 2009), and also its resolution (Dakin et al., 2015), suggesting this is an area that warrants further exploration.

This study has several limitations. One potential limitation is that the native tendon stiffness was determined in bovine tendon slices. Direct measurements of tendon stiffness in samples of healthy tendon and samples from patients at different stages of tendinopathy would have allowed us to relate our findings to tendon disease in humans. However, obtaining clinical samples of human tendon that are of sufficient size and quality to allow for AFM measurements to be made is problematic. Therefore, given the evidence that tendon structure is conserved across species, we used the bovine tendon to estimate physiological stiffness of human tendon (Lee and Elliott, 2019). The stiffness of the physiological substrate used in the study is approximately 1.2 times of that of the measured native tendon. Although the difference was not statistically significant, it is possible that this could have a minor effect on the results. The current study was limited as it only examined the effects of 2 to 2.5-fold changes in tendon matrix stiffness. It would be important to investigate the effects of greater magnitudes in matrix stiffness on TDC characteristics to develop a spectrum of responses to determine key points for targeted treatment. Also, while THP-1 cells are a validated model of macrophage-like cells, they are a monocytic cell-line derived from an acute monocytic leukemia patient, are therefore may not be truly representative of the macrophage population present in tendon. Although the THP-1results presented here are of interest, further study should look to validate these with a more representative cell population. Furthermore, the present study is limited to short time points and limited end point analyses, and therefore it is possible that important temporal dynamics between signaling responses may have been missed. The present model only tested the effect of substrate stiffness, further studies of the effect of matrix composition and architecture could provide a more comprehensive understanding about the role played by the ECM cues in regulating tendinopathy.

Overall, we established a PDMS gel based *in vitro* system that allows tailored substrate stiffness and can mimic tendon tissue. We found that TDCs and macrophages respond to changes in matrix stiffness, with softer substrate stiffness affecting TDC growth rate and gene expression, and THP-1 macrophages expressing higher levels of pro-inflammatory cytokines on substrates with non-physiological stiffness. These effects were relatively minor, and while they may contribute to the cellular changes observed in tendinopathy, it is likely that matrix stiffness, architecture and composition all work synergistically to regulate cell characteristics in healthy and tendinopathic tendon.

## Data Availability

The original contributions presented in the study are included in the article/Supplementary Material, further inquiries can be directed to the corresponding author.

## References

[B1] Abate M. Gravare-silbernagel K. Siljeholm C. Di Iorio A. De Amicis D. Salini V. (2009). Pathogenesis of Tendinopathies: Inflammation or Degeneration? Arthritis Res. Ther. 11, 235. 10.1186/ar2723 19591655 PMC2714139

[B2] Adlerz K. M. Aranda-Espinoza H. Hayenga H. N. (2016). Substrate Elasticity Regulates the Behavior of Human Monocyte-Derived Macrophages. Eur. Biophys. J. 45, 301–309. 10.1007/s00249-015-1096-8 26613613

[B3] Al-Nasiry S. Geusens N. Hanssens M. Luyten C. Pijnenborg R. (2007). The Use of Alamar Blue Assay for Quantitative Analysis of Viability, Migration and Invasion of Choriocarcinoma Cells. Hum. Reprod. 22, 1304–1309. 10.1093/humrep/dem011 17307808

[B4] Andarawis-Puri N. Flatow E. L. Soslowsky L. J. Gustave O. Place L. L. Sun H. B. (2015). Tendon Basic Science: Development, Repair, Regeneration, and Healing. J. Orthop. Res. 33, 780–784. 10.1002/jor.22869.Tendon 25764524 PMC4427041

[B5] Cetti R. Junge J. Vyberg M. (2003). Spontaneous Rupture of the Achilles Tendon Is Preceded by Widespread and Bilateral Tendon Damage and Ipsilateral Inflammation: A Clinical and Histopathologic Study of 60 Patients. Acta Orthopaedica Scand. 74, 78–84. 10.1080/00016470310013707 12635798

[B6] Chaudhuri O. Koshy S. T. Branco Da Cunha C. Shin J.-W. Verbeke C. S. Allison K. H. (2014). Extracellular Matrix Stiffness and Composition Jointly Regulate the Induction of Malignant Phenotypes in Mammary Epithelium. Nat. Mater 13, 970–978. 10.1038/nmat4009 24930031

[B7] Chen M. Zhang Y. Zhou P. Liu X. Zhao H. Zhou X. (2020). Substrate Stiffness Modulates Bone Marrow-Derived Macrophage Polarization through NF-Κb Signaling Pathway. Bioactive Mater. 5, 880–890. 10.1016/j.bioactmat.2020.05.004 PMC7332470 32637751

[B8] Chhana A. Callon K. E. Dray M. Pool B. Naot D. Gamble G. D. (2014). Interactions between Tenocytes and Monosodium Urate Monohydrate Crystals: Implications for Tendon Involvement in Gout. Ann. Rheum. Dis. 73, 1737–1741. 10.1136/annrheumdis-2013-204657 24709860

[B9] Chuang Y.-C. Chang H.-M. Li C.-Y. Cui Y. Lee C.-L. Chen C.-S. (2020). Reactive Oxygen Species and Inflammatory Responses of Macrophages to Substrates with Physiological Stiffness. ACS Appl. Mater. Inter. 12, 48432–48441. 10.1021/acsami.0c16638 33064443

[B10] Clark S. T. Zhu M. Gamble G. D. Naot D. Paine S.-J. Dalbeth N. (2020). Epidemiology of Tendon and Ligament Injuries in Aotearoa/New Zealand between 2010 and 2016. Inj. Epidemiol. 7, 1–10. 10.1186/s40621-020-0231-x 32127040 PMC7008565

[B11] Coombes B. K. Tucker K. Vicenzino B. Vuvan V. Mellor R. Heales L. (2018). Achilles and Patellar Tendinopathy Display Opposite Changes in Elastic Properties: A Shear Wave Elastography Study. Scand. J. Med. Sci. Sports 28, 1201–1208. 10.1111/sms.12986 28972291

[B12] Dakin S. G. Dudhia J. Werling N. J. Werling D. Abayasekara D. R. E. Smith R. K. W. (2012). Inflamm-Aging and Arachadonic Acid Metabolite Differences with Stage of Tendon Disease. PLoS One 7, e48978–10. 10.1371/journal.pone.0048978 23155437 PMC3498370

[B13] Dakin S. G. Martinez F. O. Yapp C. Wells G. Oppermann U. Dean B. J. F. (2015). Inflammation Activation and Resolution in Human Tendon Disease. Sci. Transl. Med. 7, 1–31. 10.1126/scitranslmed.aac4269 PMC4883654 26511510

[B14] De Fries R. Mitsuhashi M. (1995). Quantification of Mitogen Induced Human Lymphocyte Proliferation: Comparison of Alamarbluetm Assay To3h-Thymidine Incorporation Assay. J. Clin. Lab. Anal. 9, 89–95. 10.1002/jcla.1860090203 7714668

[B15] Doss B. L. Pan M. Gupta M. Grenci G. Mège R.-M. Lim C. T. (2020). Cell Response to Substrate Rigidity Is Regulated by Active and Passive Cytoskeletal Stress. Proc. Natl. Acad. Sci. USA 117, 12817–12825. 10.1073/pnas.1917555117 32444491 PMC7293595

[B16] Engler A. J. Sen S. Sweeney H. L. Discher D. E. (2006). Matrix Elasticity Directs Stem Cell Lineage Specification. Cell 126, 677–689. 10.1016/j.cell.2006.06.044 16923388

[B17] English A. Azeem A. Spanoudes K. Jones E. Tripathi B. Basu N. (2015). Substrate Topography: A Valuable *In Vitro* Tool, but a Clinical Red Herring for *In Vivo* Tenogenesis. Acta Biomater. 27, 3–12. 10.1016/j.actbio.2015.08.035 26318365

[B18] Evans J. H. Barbenel J. C. (1975). Structural and Mechanical Properties of Tendon Related to Function. Equine Vet. J. 7, 1–8. 10.1111/j.2042-3306.1975.tb03221.x 1116491

[B19] Evans N. Minelli C. Minelli C. Gentleman E. LaPointe V. Patankar S. (2009). Substrate Stiffness Affects Early Differentiation Events in Embryonic Stem Cells. eCM 18, 1–14. 10.22203/ecm.v018a01 19768669

[B20] Finnamore E. Waugh C. Solomons L. Ryan M. West C. Scott A. (2019). Transverse Tendon Stiffness Is Reduced in People with Achilles Tendinopathy: A Cross-Sectional Study. PLoS One 14, e0211863–12. 10.1371/journal.pone.0211863 30785895 PMC6382130

[B21] Fotticchia A. Musson D. Lenardi C. Demirci E. Liu Y. (2018). Anisotropic Cytocompatible Electrospun Scaffold for Tendon Tissue Engineering Elicits Limited Inflammatory Response *In Vitro* . J. Biomater. Appl. 33, 127–139. 10.1177/0885328218779846 29987990

[B22] Fu S. C. Wang W. Pau H. M. Wong Y. P. Chan K. M. Rolf C. G. (2002). Increased Expression of Transforming Growth Factor-??1 in Patellar Tendinosis. Clin. Orthopaedics Relat. Res. 400, 174–183. 10.1097/00003086-200207000-00022 12072760

[B23] Grant C. A. Brockwell D. J. Radford S. E. Thomson N. H. (2008). Effects of Hydration on the Mechanical Response of Individual Collagen Fibrils. Appl. Phys. Lett. 92, 233902. 10.1063/1.2937001

[B24] Haramshahi S. M. A. Bonakdar S. Moghtadaei M. Kamguyan K. Thormann E. Tanbakooei S. (2020). Tenocyte-imprinted Substrate: a Topography-Based Inducer for Tenogenic Differentiation in Adipose Tissue-Derived Mesenchymal Stem Cells. Biomed. Mater. 15, 035014. 10.1088/1748-605X/ab6709 31896091

[B25] Hisey C. L. Hearn J. I. Hansford D. J. Blenkiron C. Chamley L. W. (2021). Micropatterned Growth Surface Topography Affects Extracellular Vesicle Production. Colloids Surf. B: Biointerfaces 203, 111772. 10.1016/j.colsurfb.2021.111772 33894649

[B26] Hopp I. Michelmore A. Smith L. E. Robinson D. E. Bachhuka A. Mierczynska A. (2013). The Influence of Substrate Stiffness Gradients on Primary Human Dermal Fibroblasts. Biomaterials 34, 5070–5077. 10.1016/j.biomaterials.2013.03.075 23587444

[B27] Islam A. Mbimba T. Younesi M. Akkus O. (2017). Effects of Substrate Stiffness on the Tenoinduction of Human Mesenchymal Stem Cells. Acta Biomater. 58, 244–253. 10.1016/j.actbio.2017.05.058.Effects 28602855 PMC5551443

[B28] James R. Kesturu G. Balian G. Chhabra A. B. (2008). Tendon: Biology, Biomechanics, Repair, Growth Factors, and Evolving Treatment Options. J. Hand Surg. 33, 102–112. 10.1016/j.jhsa.2007.09.007 18261674

[B29] Jelinsky S. A. Archambault J. Li L. Seeherman H. (2010). Tendon-selective Genes Identified from Rat and Human Musculoskeletal Tissues. J. Orthop. Res. 28, 289–297. 10.1002/jor.20999 19780194

[B30] Kannus P. Józsa L. (1991). Histopathological Changes Preceding Spontaneous Rupture of a Tendon. A Controlled Study of 891 Patients. J. Bone Jt. Surg. 73, 1507–1525. 10.2106/00004623-199173100-00009 1748700

[B31] Kim S. J. Tatman P. D. Song D.-H. Gee A. O. Kim D.-H. Kim S. J. (2018). Nanotopographic Cues and Stiffness Control of Tendon-Derived Stem Cells from Diverse Conditions. Ijn Vol. 13, 7217–7227. 10.2147/IJN.S181743 PMC6231514 30510414

[B32] Lee A. H. Elliott D. M. (2019). Comparative Multi‐scale Hierarchical Structure of the Tail, Plantaris, and Achilles Tendons in the Rat. J. Anat. 234, 252–262. 10.1111/joa.12913 30484871 PMC6326909

[B33] Liu C. Luo J.-W. Liang T. Lin L.-X. Luo Z.-P. Zhuang Y.-Q. (2018). Matrix Stiffness Regulates the Differentiation of Tendon-Derived Stem Cells through FAK-Erk1/2 Activation. Exp. Cel Res. 373, 62–70. 10.1016/j.yexcr.2018.08.023 30138615

[B34] Liu F. Mih J. D. Shea B. S. Kho A. T. Sharif A. S. Tager A. M. (2010). Feedback Amplification of Fibrosis through Matrix Stiffening and COX-2 Suppression. J. Cel Biol. 190, 693–706. 10.1083/jcb.201004082 PMC2928007 20733059

[B35] Mao Z. Fan B. Wang X. Huang X. Guan J. Sun Z. (2021). A Systematic Review of Tissue Engineering Scaffold in Tendon Bone Healing *In Vivo* . Front. Bioeng. Biotechnol. 9, 621483. 10.3389/fbioe.2021.621483 33791283 PMC8005599

[B36] Mih J. D. Marinkovic A. Liu F. Sharif A. S. Tschumperlin D. J. (2012). Matrix Stiffness Reverses the Effect of Actomyosin Tension on Cell Proliferation. J. Cel Sci. 125, 5974–5983. 10.1242/jcs.108886 PMC3585515 23097048

[B37] Millar N. L. Gilchrist D. S. Akbar M. Reilly J. H. Kerr S. C. Campbell A. L. (2015). MicroRNA29a Regulates IL-33-mediated Tissue Remodelling in Tendon Disease. Nat. Commun. 6, 6774. 10.1038/ncomms7774 25857925 PMC4403384

[B38] Millar N. L. Hueber A. J. Reilly J. H. Xu Y. Fazzi U. G. Murrell G. A. C. (2010). Inflammation Is Present in Early Human Tendinopathy. Am. J. Sports Med. 38, 2085–2091. 10.1177/0363546510372613 20595553

[B39] Morita W. Snelling S. J. B. Dakin S. G. Carr A. J. (2016). Profibrotic Mediators in Tendon Disease: A Systematic Review. Arthritis Res. Ther. 18, 1–11. 10.1186/s13075-016-1165-0 27863509 PMC5116130

[B40] Musson D. S. Naot D. Chhana A. Matthews B. G. McIntosh J. D. Lin S. T. C. (2015). *In Vitro* Evaluation of a Novel Non-Mulberry Silk Scaffold for Use in Tendon Regeneration. Tissue Eng. A 21, 1539–1551. 10.1089/ten.tea.2014.0128 PMC4426299 25604072

[B41] Nichols A. E. C. Best K. T. Loiselle A. E. (2019). The Cellular Basis of Fibrotic Tendon Healing: Challenges and Opportunities. Translational Res. 209, 156–168. 10.1016/j.trsl.2019.02.002 PMC6545261 30776336

[B42] Paszek M. J. Zahir N. Johnson K. R. Lakins J. N. Rozenberg G. I. Gefen A. (2005). Tensional Homeostasis and the Malignant Phenotype. Cancer Cell 8, 241–254. 10.1016/j.ccr.2005.08.010 16169468

[B43] Petersen W. Stein V. Bobka T. (2000). Structure of the Human Tibialis Anterior Tendon. J. Anat. 197, 617–625. 10.1017/S002187829900696210.1046/j.1469-7580.2000.19740617.x 11197535 PMC1468177

[B44] Previtera M. L. Sengupta A. (2015). Substrate Stiffness Regulates Proinflammatory Mediator Production through TLR4 Activity in Macrophages. PLoS One 10, e0145813. 10.1371/journal.pone.0145813 26710072 PMC4692401

[B45] Riley G. P. Harrall R. L. Constant C. R. Chard M. D. Cawston T. E. Hazleman B. L. (1994). Tendon Degeneration and Chronic Shoulder Pain: Changes in the Collagen Composition of the Human Rotator Cuff Tendons in Rotator Cuff Tendinitis. Ann. Rheum. Dis. 53, 359–366. 10.1136/ard.53.6.359 8037494 PMC1005350

[B46] Ryan C. N. M. Pugliese E. Shologu N. Gaspar D. Rooney P. Islam M. N. (2021). A Combined Physicochemical Approach towards Human Tenocyte Phenotype Maintenance. Mater. Today Bio 12, 100130. 10.1016/j.mtbio.2021.100130 PMC8488312 34632361

[B47] Sharma R. I. Snedeker J. G. (2010). Biochemical and Biomechanical Gradients for Directed Bone Marrow Stromal Cell Differentiation toward Tendon and Bone. Biomaterials 31, 7695–7704. 10.1016/j.biomaterials.2010.06.046 20656345

[B48] Skardal A. Mack D. Atala A. Soker S. (2013). Substrate Elasticity Controls Cell Proliferation, Surface Marker Expression and Motile Phenotype in Amniotic Fluid-Derived Stem Cells. J. Mech. Behav. Biomed. Mater. 17, 307–316. 10.1016/j.jmbbm.2012.10.001 23122714 PMC3665276

[B49] Sridharan R. Cavanagh B. Cameron A. R. Kelly D. J. O'Brien F. J. (2019). Material Stiffness Influences the Polarization State, Function and Migration Mode of Macrophages. Acta Biomater. 89, 47–59. 10.1016/j.actbio.2019.02.048 30826478

[B50] Starr T. Bauler T. J. Malik-Kale P. Steele-Mortimer O. (2018). The Phorbol 12-Myristate-13-Acetate Differentiation Protocol Is Critical to the Interaction of THP-1 Macrophages with Salmonella Typhimurium. PLoS One 13, e0193601–13. 10.1371/journal.pone.0193601 29538403 PMC5851575

[B51] Stolk M. Klatte-Schulz F. Schmock A. Minkwitz S. Wildemann B. Seifert M. (2017). New Insights into Tenocyte-Immune Cell Interplay in an *In Vitro* Model of Inflammation. Sci. Rep. 7, 1–14. 10.1038/s41598-017-09875-x 28851983 PMC5575127

[B52] Taylor S. E. Vaughan-Thomas A. Clements D. N. Pinchbeck G. MacRory L. C. Smith R. K. (2009). Gene Expression Markers of Tendon Fibroblasts in normal and Diseased Tissue Compared to Monolayer and Three Dimensional Culture Systems. BMC Musculoskelet. Disord. 10, 1–10. 10.1186/1471-2474-10-27 19245707 PMC2651848

[B53] Vasiliadis A. V. Katakalos K. (2020). The Role of Scaffolds in Tendon Tissue Engineering. Jfb 11, 78–11. 10.3390/jfb11040078 PMC7712651 33139620

[B54] Wang J. H.-C. H. (2006). Mechanobiology of Tendon. J. Biomech. 39, 1563–1582. 10.1016/j.jbiomech.2005.05.011 16000201

[B55] Wang Z. Volinsky A. A. Gallant N. D. (2014). Crosslinking Effect on Polydimethylsiloxane Elastic Modulus Measured by Custom-Built Compression Instrument. J. Appl. Polym. Sci. 131, a–n. 10.1002/app.41050

[B56] Whittaker P. Canham P. B. (1991). Demonstration of Quantitative Fabric Analysis of Tendon Collagen Using Two-Dimensional Polarized Light Microscopy. Matrix 11, 56–62. 10.1016/S0934-8832(11)80227-1 2027329

[B57] Wiesinger H.-P. Seynnes O. R. Kösters A. Müller E. Rieder F. (2020). Mechanical and Material Tendon Properties in Patients with Proximal Patellar Tendinopathy. Front. Physiol. 11, 1–11. 10.3389/fphys.2020.00704 32733263 PMC7358637

[B58] Xing X. Wang Y. Zhang X. Gao X. Li M. Wu S. (2021). Matrix Stiffness‐mediated Effects on Macrophages Polarization and Their LOXL2 Expression. FEBS J. 288, 3465–3477. 10.1111/febs.15566 32964626

[B59] Yao L. Bestwick C. S. Bestwick L. A. Maffulli N. Aspden R. M. (2006). Phenotypic Drift in Human Tenocyte Culture. Tissue Eng. 12, 1843–1849. 10.1089/ten.2006.12.1843 16889514

[B60] Zhang C. Tan Y. Feng J. Huang C. Liu B. Fan Z. (2020). Exploration of the Effects of Substrate Stiffness on Biological Responses of Neural Cells and Their Mechanisms. ACS Omega 5, 31115–31125. 10.1021/acsomega.0c04279 33324820 PMC7726759

[B61] Zhu M. F. Smith B. Krishna S. Musson D. S. Riordan P. R. McGlashan S. R. (2020). The Pathological Features of Hip Abductor Tendon Tears - a Cadaveric Study. BMC Musculoskelet. Disord. 21, 1–9. 10.1186/s12891-020-03784-3 PMC7690166 33243210

