# Supplementary material for: Changes in Physiological Tendon Substrate Stiffness Have Moderate Effects on Tendon-Derived Cell Growth and Immune Cell Activation
Source: Front Bioeng Biotechnol. 2022 Feb 28;10:800748. doi: 10.3389/fbioe.2022.800748 (PMC8918575; doi:10.3389/fbioe.2022.800748)
Supplement: Supplementary file 1 [file DataSheet1.PDF]

Supplementary Material

Supplementary Table 1 Details of tendon donors and characteristics evaluated

Sex	Age	Tissue source	Characteristics evaluated		
			Cell growth	Cell morphology	Gene expression profile
Female	48	Hamstring	X	X	X
Female	48	Hamstring	X		
Male	46	Hamstring	X	X	X
Male	50	Biceps	X		X
Male	51	Biceps	X	X	X

Supplementary Table 2 Genes studied by RT-real time PCR

Gene symbol	Gene Name	Relevance to the study	TaqMan® assay
SCX	Scleraxis	Key regulator of tendon cell fate (Taylor et al., 2009)	Hs03054634_m1
THBS4	Thrombospondin-4	Tendon selective gene (Jelinsky et al., 2010)	Hs00170261_m1
TNC	Tenascin-C	Tendon glycoprotein (Taylor et al., 2009)	Hs01115665_m1
COL1A1	Collagen-I	Major collagen of tendon (Riley et al., 1994)	Hs00164004_m1
COL3	Collagen-III	Second most abundant collagen in tendon (Riley et al., 1994)	Hs00943809_m1

MMP3	Matrix Metalloproteinase- 3	Matrix degradation enzyme(Arnoczky et al., 2007)	Hs00968305_m1
SOX9	SRY-Box Transcription Factor 9	Gene associated with chondrocyte differentiation (Haseeb et al., 2021)	Hs00165814_m1
CTGF	Connective tissue growth Factor	Fibrosis associated gene(Lipson et al., 2012)	Hs00170014_m1
ALPL	Alkaline phosphatase	Gene associated with osteoblast differentiation(Anh et al., 1998)	Hs00602161_m1
TGFB1	Transforming Growth Factor- β	Pro-healing factor produced by macrophage(Hesketh et al., 2017)	Hs00998133_m1
IL8	Interlukin-8	Inflammatory cytokine produced by macrophages(Hesketh et al., 2017)	Hs00174103_m1
IL1B	Interlukin-1β	Inflammatory cytokine produced by macrophage(Hesketh et al., 2017)	Hs01555410_m1

Supplementary Figures

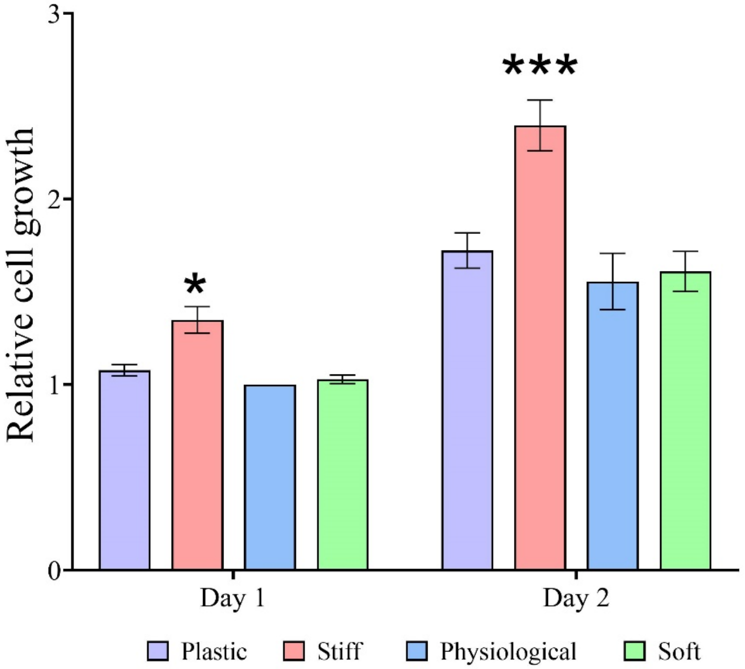

Supplementary Figure 1. MC3T3 growth on substrates with different stiffness. Cell growth was determined by alamarBlue™ assay. Results are presented as means ±SEM (n=4). Groups were compared by two-way ANOVA with post-hoc Dunnett's test.

*$p<0.05$, ***$p<0.001$ in comparison to cell growth on substrate with physiological stiffness on day 1.
